# Indication for biologic treatment in a real-world cohort of chronic rhinosinusitis patients according to international recommendations: Evidence from the European CRS outcome registry (CHRINOSOR)^☆^

**DOI:** 10.1016/j.waojou.2026.101365

**Published:** 2026-03-23

**Authors:** Sven F. Seys, Josje J. Otten, Joost de Kinderen, Giulia Bettio, Alexandros Andrianakis, Isam Alobid, Carlo Cavaliere, Peter W. Hellings, Laura Van Gerven, Valérie Hox, Claire Hopkins, Anette Kjeldsen, Sven Schneider, Peter-Valentin Tomazic, Zuzana Diamant, Julia Eckl-Dorna, Wytske J. Fokkens, Clemens Holzmeister, Kenneth Larsen, Anu Laulajainen-Hongisto, Valerie Lund, Gert Mariën, Simonetta Masieri, Geoffrey Mortuaire, Mathilde Moyaert, Joaquim Mullol, Camilo Rodriguez van Strahlen, Marco de Vincentiis, Martin Wagenmann, Sietze Reitsma, Claus Bachert

**Affiliations:** a Galenus Health (part of Cascador Health), Belgium; b Department of Otorhinolaryngology, Amsterdam University Medical Centres (AUMC), location AMC, Amsterdam, the Netherlands; c Department of General ORL, Head and Neck Surgery, Medical University of Graz, Graz, Austria; d Rhinology Unit & Smell Clinic and Skull Base Surgery Unit, Otorhinolaryngology Department, Hospital Clinic Barcelona, Universitat de Barcelona, IDIBAPS, CIBERES, Barcelona, Catalonia, Spain; e Department of Sense Organs, Sapienza University, Rome, Italy; f Department of Otorhinolaryngology, Head and Neck Surgery, University Hospitals Leuven, Leuven, Belgium; g Allergy and Clinical Immunology Research Group, Department of Microbiology, Immunology & Transplantation, KU Leuven, Belgium; h Department of Neurosciences, Experimental Otorhinolaryngology, Rhinology Research, KU Leuven, Leuven, Belgium; i Service d'Otorhinolaryngologie, Cliniques Universitaires Saint-Luc, Brussels, Belgium; j ENT Department, Guy's and St Thomas' NHS Foundation Trust, London, United Kingdom; k Department of Otorhinolaryngology, Odense University Hospital, Odense, Denmark; l Department of Otorhinolaryngology, Head and Neck Surgery, Vienna General Hospital (AKH), Medical University of Vienna, Vienna, Austria; m Department of Respiratory Medicine, First Faculty of Medicine, Charles University and Thomayer Hospital, Prague, Czech Republic; n Univ Groningen, Univ Med Ctr Groningen, Dept Clin Pharm & Pharmacol, Netherlandsof Clin Pharm & Pharmacol, University Groningen, University Medical Center Groningen, Groningen, Netherlands; o Department of Otorhinolaryngology-Head and Neck Surgery, Helsinki University Hospital and University of Helsinki, Finland; p Department of Rhinology, Royal National Ear, Nose, Throat and Eastman Dental Hospital, UCLH, United Kingdom; q Department of Oral and Maxillofacial Sciences, Sapienza University, Rome, Italy; r Otorhinolaryngology-Head and Neck Department, Huriez Hospital, Centre Hospitalier Universitaire (CHU) Lille, Lille, France; s Department of Otorhinolaryngology, Universitätsklinikum Düsseldorf (UKD), Dusseldorf, Germany; t Clinic for ENT diseases and Head and Neck Surgery, University Clinic Münster, Münster, Germany; u Department of Otorhinolaryngology, First Affiliated Hospital, Sun Yat-Sen University, International Airway Research Center, Guangzhou, China

**Keywords:** Chronic rhinosinusitis, nasal polyps, biologic, Indication, Real-world evidence

## Abstract

**Background:**

In recent years, treatment options for patients with uncontrolled severe chronic rhinosinusitis with nasal polyps (CRSwNP) have been expanded with biologics. Definitions of uncontrolled severe disease differ across international recommendations and the prescription of biologics depends on national reimbursement criteria.

**Objective:**

We aimed to evaluate the indication for biologic treatment, based on international recommendations, in a real-world cohort of CRSwNP patients.

**Methods:**

CRS patients who visited the outpatient ENT clinic of 10 tertiary centres from 7 European countries were invited to use the Galenus Health mobile application monitoring their disease. The proportion of patients who fulfilled biologic indication criteria according to EUFOREA 2021 and EPOS/EUFOREA 2023 recommendations was evaluated.

**Results:**

A total of 281 CRS patients were recruited of which 227 (82.1%) were diagnosed as CRSwNP. Out of these 227 patients, 21 patients with prior biologic use were excluded, resulting in a cohort of 206 patients. A total of 28.7% (50/174) and 47.6% (81/170) of CRSwNP patients, met the EUFOREA 2021 or EPOS/EUFOREA 2023 criteria for indication for biologic treatment, respectively. Biologic treatment was initiated at the time of inclusion in the cohort in 18.9% (39/206) of CRSwNP patients.

**Conclusion:**

According to international recommendations, 29–48% of CRSwNP patients in tertiary centres may be a candidate for biologic treatment. EPOS/EUFOREA2023 criteria were more inclusive than EUFOREA 2021 criteria with respect to biologic indication for CRSwNP. National reimbursement criteria prevail over international recommendations for actual biologic prescription and are less restrictive in some countries.

## Introduction

For several decades, for the majority of patients with chronic rhinosinusitis (CRS) intranasal corticosteroids (INCS) and saline irrigation have been the initial appropriate medical treatment (AMT), followed by endoscopic sinus surgery (ESS) or short courses of systemic corticosteroids (SCS) in those that failed AMT.^1^ SCS in particular are associated with severe potential adverse effects.^2^ More recently, biologics were added to the treatment options for CRS patients with bilateral nasal polyps (CRSwNP).^3^ Five different biologics (benralizumab, dupilumab, mepolizumab, omalizumab, and tezepelumab) have been evaluated in phase III clinical trials, showing varying effects on nasal polyp score (NPS), CRS-related symptoms, and on quality of life.^4^, ^5^, ^6^, ^7^, ^8^ Dupilumab was the first biologic marketed for CRSwNP, followed by omalizumab and mepolizumab and more recently tezepelumab.^9^ Real-world studies also demonstrated effectiveness of dupilumab, mepolizumab and omalizumab for CRSwNP.^10^, ^11^, ^12^, ^13^, ^14^, ^15^, ^16^

According to the EPOS 2020 guidelines, biologic treatment was indicated in patients with a history of prior sinus surgery and when 3 out of 5 of the following criteria could be documented: evidence of type 2 inflammation (tissue eosinophils ≥ 10 cells/high power field and/or blood eosinophil counts ≥ 250 cells/μ L and/or total IgE ≥ 100 IU/ml), need for SCS (≥ 2 courses of SCS per year or long-term low dose SCS), significantly impaired quality of life (SNOT-22 ≥ 40), significant loss of smell (anosmia confirmed by a smell test) and/or presence of comorbid asthma (requiring regular inhaled corticosteroids (ICS).^3^

Based on new insights, indication criteria for biologics have been adapted over the past years.^17^^,^^18^ According to the EUFOREA 2021 recommendations, patients with uncontrolled severe bilateral CRSwNP have an indication for biologic treatment.^17^ Uncontrolled disease was defined as persistent or recurring CRS with nasal polyps (CRSwNP) despite long-term INCS and if they had received ≥ 1 course of SCS in the preceding 2 years and/or previous sinonasal surgery; severe disease was defined as bilateral CRSwNP with a NPS of ≥ 4 out of 8 points and persistent symptoms with the need for add-on treatment to supplement INCS. Specific cutoff values were proposed for the presence of persistent symptoms: loss of smell score (0–3) ≥ 2, nasal congestion score (0–3) ≥ 2, SNOT-22 score ≥ 35, total symptoms visual analogue scale (VAS) ≥ 50 mm. In the subsequent EPOS/EUFOREA 2023 update, the cutoff for blood eosinophil counts was lowered from 250 to 150 cells/μL compared to EPOS 2020, whereas other cutoff points remained unaltered.^18^

Apart from indication criteria proposed by opinion leader consortia, national reimbursement criteria mainly determine the actual patient prescription of biologics. Although these criteria are related to the international recommendations, they are not identical. For example, cutoff values may differ from international recommendations, or the absence of pre-set cutoff values refers to the interpretation of the treating physician.

In the current study, we evaluated indication for biologics according to the aforementioned international recommendations and their actual prescription in a real-world CRS cohort using data from the Galenus Health mobile application (app).

## Methods

### Study design and population

This was an observational, cross-sectional, multicentre study conducted in 10 tertiary centres from 7 European countries: Austria (n = 28 patients), Belgium (n = 51 patients), Denmark (n = 18 patients), Italy (n = 14 patients), Spain (n = 71 patients), the Netherlands (n = 1 patients), and the United Kingdom (n = 13 patients). Data was collected between 2021 and 2024 to establish a cohort of prospectively collected data from CRS patients who visited the outpatient clinic of the participating centres in the real-world setting and who were invited to use the Galenus Health app^19^ for the follow-up of their disease. The study was approved by the local institutional review boards (Table S2), except for the Netherlands (not required according to the Dutch Medical Research Involving Human Subject Act), UK and Denmark (not required according to national legislation) and registered at clinicaltrials.gov (NCT04670172).

### Inclusion and exclusion criteria

In order to reflect the real-life CRS population as much as possible ('’unselected cohort’’), the number of exclusion criteria were kept to a minimum. Adult patients with CRS diagnosis according to EPOS 2020 and with a known NP status were included in the unselected cohort. Patients with CRS due to malignancies, inverted papilloma and unilateral disease, as well as patients which were already on a biologic therapy prior to the start of using the Galenus Health app, were excluded. All patients were required to use the Galenus Health app for data collection.

### Data collection and outcome measures

Patients were invited to complete their health profile at the start of using the Galenus Health app. As previously described,^19^ the health profile consisted of an electronic questionnaire to retrieve information on the patient's demographic characteristics (age, gender, body mass index (BMI), smoking history), disease history (number of courses of SCS in the past year, number of endoscopic sinus surgery [ESS] ever), presence of comorbidities (NSAID exacerbated respiratory disease [NERD], asthma, allergy). Patients were asked to complete the health diary using the Galenus Health app and to provide information on total and specific CRS-related symptoms by visual analogue scale (VAS) (from 0 “no burdensome symptoms” to 100 “extremely burdensome symptoms”) on a weekly basis. Disease-specific health-related questionnaires were completed at the start of using the app and patients received a notification to complete the questionnaires each month: Sino-Nasal Outcome Test-22 (SNOT-22) on a total score of 110, Asthma Control Test (ACT) on total score of 25. Additionally, the following clinical parameters were assessed during hospital visit and were retrieved from the (electronic) medical records of the patient: nasal polyp score (NPS: 0–4 on every side, total score 0–8), Lund-Mackay score (0–24), blood eosinophil counts (BEC), and serum total IgE level. We here report on data collected at time of onboarding the mobile application during hospital outpatient visit (index date).

### Evaluation of indication for biologic therapy

Both the EUFOREA 2021 criteria^17^ and the EPOS/EUFOREA 2023 criteria^18^ were applied to assess potential indication of CRS patients for biologic treatment. Available real-world outcomes were assessed in line with the parameters mentioned in Table 1.

**Table 1:** **Criteria for biologic indication in CRSwNP patients based on international recommendations**.

EUFOREA 2021	Real-world measured
Chronic rhinosinusitis	Physician-diagnosed

Bilateral nasal polyps	Physician-diagnosed

At least 1 course of SCS in the past 2 years	Number of SCS courses in the past year

Previous sinonasal surgery	History of endoscopic sinus surgery

Nasal polyp score ≥ 4	Nasal polyp score ≥ 4

Loss of smell score ≥ 2	VAS loss of smell ≥ 52 mm∗

Nasal congestion score ≥ 2	VAS nasal blockage ≥ 50 mm

Total symptom VAS ≥ 50 mm	VAS total sinus symptoms ≥ 50 mm

SNOT-22 ≥ 35	SNOT-22 ≥ 35

EPOS/EUFOREA 2023	Real-world measured
Chronic rhinosinusitis	Physician-diagnosed
Bilateral nasal polyps	Physician-diagnosed
Previous sinonasal surgery	History of endoscopic sinus surgery
Evidence of type 2 inflammation (BEC ≥ 150 cells/μl and/or serum total IgE ≥ 100 IU/ml)	BEC ≥ 150 cells/μl and/or serum total IgE ≥ 100 IU/ml
At least 2 course of SCS in the past year	Number of SCS courses in the past year
SNOT-22 ≥ 40	SNOT-22 ≥ 40
Anosmic on a smell test	VAS loss of smell ≥ 52 mm∗
Asthma requiring ICS	Physician-diagnosed

EUFOREA 2021 criteria from Bachert et al.^17^ and EPOS/EUFOREA 2023 criteria from Fokkens et al.^18^ Criteria applied in the real-world setting and reported in this manuscript can be found in the second column. Since not all of the criteria were measured in the real-world setting, surrogate measurements have been proposed. ∗: Adapted cutoff value in line with Alobid et al. and Otten et al..^24^^,^^25^ SCS: systemic corticosteroids, SNOT-22: sinonasal outcome test-22, ICS: inhaled corticosteroids

### Statistics

Descriptive statistics were applied to analyse the provided data. Fisher's exact test was used for comparison of proportions between groups. A p value ≤ 0.05, two-sided, was considered significant. Statistical analyses were performed using Graphpad Prism version 10.

## Results

### Patient characteristics

Out of 281 CRS patients, 274 had a known NP status, of which 82.8% (227/274) were diagnosed with CRSwNP and were included. 21 CRSwNP patients had previously started on a biologic treatment and were therefore excluded for this analysis, which resulted in a study cohort of 206 patients. 39 (18.9%) patients started treatment with a biologic for the primary indication of CRSwNP at day of inclusion in the cohort (dupilumab: n = 27, mepolizumab: n = 10, omalizumab: n = 2). The characteristics of the study cohort of 206 patients at time of enrolment are presented in Table 2. Approximately half of patients (52.2%) had received SCS in the past year. A majority of patients had a history of ESS (68.1%). Type 2 inflammation was present in 89.0% of patients with 81.7% showing BEC ≥ 150 cells/μl and 44.3% showing serum total IgE levels ≥ 100 IU/ml. 59.7% of patients had a self-reported diagnosis of comorbid asthma.

**Table 2:** **Patient characteristics of the CRSwNP cohort at time of enrolment**.

Patient characteristics	CRSwNP cohort (n = 206)
Male – Female ratio, (%)	60.0–40.0
Age in years, mean ± SD	50.5 ± 12.1
BMI in kg/m^2^, mean ± SD	26.0 ± 4.8
Smoking status (current-ex-never smoker), (%)	13.3–35.2 – 51.6
Allergy, (%)	59.5
Asthma, (%)	59.7
NERD, (%)	24.0
CRS disease duration in years, median (IQR)	11.0 (4.0–21.0)
Number of ESS procedures (0-1-2-3->3), (%)	31.9–39.4 – 13.8–8.5 – 6.4
SCS courses past year (0-1-2-3->3), (%)	47.8–19.8 – 15.4–7.7 – 9.3
Intranasal corticosteroids,%	50.3
Inhaled corticosteroids,%	38.0
Biologics therapy,%	18.9
SNOT-22 score, median (IQR)	37.0 (18.5–59.0)
Lund-Mackay score, median (IQR)	14.5 (9.0–20.0)
VAS total sinus symptoms in mm, median (IQR)	41.0 (14.5–68.5)
VAS nasal blockage in mm, median (IQR)	33.0 (8.0–65.3)
VAS loss of smell in mm, median (IQR)	62.5 (14.5–98.8)
NPS (0/2–3/4 - 5/6–7/8), (%)	45.6–30.4 – 21.5–2.5
Blood eosinophil counts in cells/μl, median (IQR)	379.0 (200.0–600.0)
Serum total IgE levels in IU/ml, median (IQR)	77.0 (17.0–211.5)
Type 2 inflammation,%	89.0
Blood eosinophil counts ≥150 cells/μl,%	81.7
Serum total IgE levels ≥100 IU/ml,%	44.3

Age and BMI were represented as mean ± standard deviation. SNOT-22 score, Lund-Mackay score, visual analogue scale (VAS), blood eosinophil counts and serum total IgE levels were represented as median with interquartile range. BMI: body mass index, CRS: chronic rhinosinusitis, NP: nasal polyps, NERD: NSAID exacerbated respiratory disease, ESS: endoscopic sinus surgery, SCS: systemic corticosteroids, SNOT-22: sinonasal outcome test-22, NPS: nasal polyp score

### Uncontrolled severe disease based on different international recommendations

#### EUFOREA 2021 criteria

The proportion of patients meeting the individual EUFOREA 2021 criteria are listed in Table 4. 83.6% (163/195 patients) received ≥1 course of SCS in the past year (54.8%, 103/188), and/or previous ESS (71.3%, 139/195). 34.7% (58/167) of CRSwNP patients had severe disease based on a NPS ≥ 4 (44.3%, 70/158), and persistent symptoms as measured by VAS LoS ≥ 52 mm (54.1%, 105/194), VAS NB ≥ 50 mm (37.2%, 73/196), VAS total sinus symptoms ≥ 50 mm (43.2%, 86/199) or SNOT-22 ≥ 35 (53.4%, 102/191). 28.7% (50/174) of CRSwNP patients met EUFOREA 2021 criteria for uncontrolled severe disease (Table 3). The proportion of CRSwNP patients who met these criteria were similar between patients who initiated biologic treatment at time of inclusion (31.4%, 11/35) and those who did not (28.1%, 39/139), p > 0.99; Table 3. The percentage of patients meeting the indication criteria of EUFOREA 2021 but not (yet) receiving biologics was 78.0% (39/50 patients).

**Table 3:** **Comparison of uncontrolled severe disease status between international recommendations**.

Assessment based EUFOREA 2021 criteria		
	Criteria met	Criteria not met
Not on biologics,%	28.1 (39/139)	71.9 (100/139)
On biologics,%	31.4 (11/35)	63.2 (24/35)
Total,%	28.7 (50/174)	71.3 (124/174)

**Assessment based EPOS/EUFOREA 2023 criteria**		
	Criteria met	Criteria not met

Not on biologics,%	43.1 (59/137)	56.9 (78/137)
On biologics,%	66.7 (22/33)	33.3 (11/33)
Total,%	47.6 (81/170)	52.4 (89/170)

Patients who started with a biologic treatment at the day of the analysis were reported separately

**Table 4:** **Biologic indication of CRSwNP patients according to EUFOREA 2021 criteria**.

EUFOREA 2021 CRITERIA		Criteria met,%	
History	History of ESS	**71.3**	(139/195)
	At least 1 SCS course last year	**54.8**	(103/188)
	History of ESS and/or at least 1 SCS course last year	**83.6**	(163/195)
Severe	NPS ≥ 4 points	**44.3**	(70/158)
Uncontrolled	SNOT-22 ≥ 35 points	**53.4**	(102/191)
	VAS TSS ≥ 50 mm	**43.2**	(86/199)
	VAS NB ≥ 50 mm	**37.2**	(73/196)
	VAS LoS ≥ 52 mm	**54.1**	(105/194)
	Meets the SNOT-22 criteria and/or any of the 3 VAS criteria	**75.7**	(156/206)
Uncontrolled severe	NPS ≥ 4 and meets at least 1 uncontrolled criteria	**34.7**	(58/167)
**Criteria met**	**History plus uncontrolled severe**	**28.7**	(50/174)

CRSwNP: chronic rhinosinusitis with nasal polyps, ESS: endoscopic sinus surgery, SCS: oral corticosteroids, SNOT-22: sinonasal outcome test-22, NPS: nasal polyp score, VAS: visual analogue scale, TSS: total sinus symptoms, NB: nasal blockage, LoS: loss of smell

#### EPOS/EUFOREA 2023 criteria

The proportion of patients meeting the individual EUFOREA 2021 criteria are listed in Table 5. 71.3% (139/195) reported a history of ESS. Five other criteria determined whether a patient may be considered “uncontrolled severe” and thus indicated for biologic treatment: ie, the presence of type-2 inflammation (90.3%, 139/154), defined by BEC ≥ 150 cells/μ L (81.7%, 125/153) and/or serum total IgE ≥ 100 IU/ml (44.3%, 66/149), ≥2 courses of SCS in the past year (33.0%, 60/182), SNOT-22 score ≥ 40 (46.1%, 88/191), VAS LoS ≥ 52 mm (54.1, 105/194), and the presence of comorbid asthma (60.8%, 124/204). At least 3 out of 5 of the abovementioned criteria for uncontrolled severe disease were met by 67.7% (105/155) of patients. 47.6% (81/170) of CRSwNP patients met EPOS/EUFOREA 2023 criteria for indication of biologic treatment (Table 3). The proportion of CRSwNP patients who met these criteria were higher in patients who were prescribed a biologic at time of inclusion (66.7%, 22/33) than those who are not (43.1%, 59/137), p = 0.02; Table 3. The percentage of patients meeting the indication criteria but not (yet) receiving biologics was 72.8% (59/81 patients) according to the EPOS/EUFOREA 2023 recommendations.

**Table 5:** **Biologic indication of CRSwNP patients according to EPOS/EUFOREA 2023 criteria**.

EPOS/EUFOREA 2023 CRITERIA		Criteria met,%	
History	History of ESS	**71.3**	(139/195)
Blood values	BEC ≥ 150 cells/ul	**81.7**	(125/153)
	Serum total IgE ≥ 100 IU/ml	**44.3**	(66/149)
Must meet 3 out of 5 criteria for uncontrolled severe	Type-2 (BEC ≥ 150 and/or serum total IgE ≥ 100)	**90.3**	(139/154)
	At least 2 SCS courses last year	**33.0**	(60/182)
	Impaired QoL measured by SNOT-22 ≥ 40 points	**46.1**	(88/191)
	Impaired smell measured by VAS LoS ≥ 52 mm	**54.1**	(105/194)
	Asthma	**60.8**	(124/204)
	Meets at least 3 out of the following 5: Type-2 and/or ≥ 2 SCS courses and/or impaired QoL and/or impaired smell and/or asthma	**67.7**	(105/155)
**Criteria met**	**Prior surgery and meets 3 out of 5 uncontrolled severe criteria**	**47.6**	**(81/170)**

CRSwNP: chronic rhinosinusitis with nasal polyps, ESS: endoscopic sinus surgery, SCS: systemic corticosteroids, SNOT-22: sinonasal outcome test-22, NPS: nasal polyp score, VAS: visual analogue scale, TSS: total sinus symptoms, NB: nasal blockage, LoS: loss of smell

### Comparison of international recommendations

There were 74.3% (153/206) of patients who had sufficient data to evaluate them according to both international recommendations. Agreement between EUFOREA 2021 and EPOS/EUFOREA 2023 recommendations was observed in 62.1% (95/153 of patients, classifying patients in meeting (20.3%, 31/153) or not meeting (41.8, 64/153) biologic indication criteria for both recommendations. Discordance was observed in 37.9% (58/153) of patients, classifying patients in meeting only EUFOREA 2021 criteria (11.1%, 17/153) or meeting only EPOS/EUFOREA 2023 criteria (26.8%, 41/153). Nasal polyp score was found to be the most limiting factor (44.3% with NPS ≥ 4) in EUFOREA 2021.

## Discussion

This real-world study evaluated and compared 2 international recommendations (EUFOREA 2021 and EPOS/EUFOREA 2023) defining uncontrolled severe CRSwNP patients with an indication for biological treatment. Our findings indicate that 28.7% of CRS patients in an unselected multicentre cohort met the EUFOREA 2021 criteria, while 47.6% met the updated EPOS/EUFOREA 2023 criteria. This 18.8% difference in meeting indication criteria is due to differences in the construction of the criteria sets, and may, in part, arise from the requirement of a nasal polyp score (NPS) threshold of ≥4 in the 2021 recommendations.^18^ As the NPS is assessed by physicians, it is subject to variability in interpretation.^20^ Additionally, factors such as recent SCS use or endoscopic sinus surgery (ESS) can significantly affect the NPS. As a result, some patients who may actually benefit from biological treatment might not qualify under the EUFOREA 2021 criteria. Another notable difference between the 2 recommendations is the cutoff value of the SNOT-22 score of ≥35 in the 2021 recommendations versus ≥40 in the 2023 recommendations. When applying the 2021 recommendations, 53.4% of our cohort met this “significantly impaired quality of life” criterion compared to 46.1% when applying the 2023 recommendations. Lastly, EUFOREA 2021 criteria refer to SCS use in the proceeding 2 years whereas in our study only data on SCS use of the past year could be analysed.

There is a marked discrepancy between the number of patients meeting indication criteria and the number of patients who were actually prescribed biological treatment (78.0% (39/50) according to EUFOREA 2021 and 72.8% (59/81) according to EPOS/EUFOREA 2023). These numbers are somewhat aligned applying either of the international recommendations and reflect the dominance of national reimbursement criteria for actual biologic prescription (as outlined in Table S1) or the prescribing behaviour of medical specialists. For example, in the Netherlands, only non-smokers are eligible for biological treatment. In quite a few European countries, the bilateral NPS has to be ≥ 5 (Table S 1). Additionally, the SNOT-22 cutoff is set at ≥50 in a number of cases (both these cutoff values apply eg, in Italy and Denmark). To complicate matters further, such national criteria can be subject to change, in an effort to control costs. Until cost-effectiveness of biological treatment in Europe is established,^21^^,^^22^ more inclusive recommendations would be less attractive from a cost perspective. In addition to that, the percentage of patients meeting the EUFOREA or EPOS/EUFOREA 2023 criteria but not receiving a biologic are calculated at a single point in time. Some patients might receive biologics at a later stage. This makes the abovementioned percentages prone to overestimation. Treating physicians may have preferences for other therapeutic options for various reasons (including but not limited to awareness of biologics, its effectiveness and applicable indication criteria, or may have concerns about adverse events or prefer less costly therapy options). Of note is also the joint decision-making with other physicians in case of comorbidities. Lastly, patients who meet indication criteria can still also prefer other treatment options like ESS.

Another discrepancy found is the percentage of patients who did receive biologic treatment despite not meeting the international criteria (63.2% according to EUFOREA 2021 versus 33.3% according to EPOS/EUFOREA 2023 in Table 3). The difference in percentages between these international criteria might be explained by the fact that the EPOS/EUFOREA 2023 guidelines are more inclusive, as mentioned before. Again, also for these patients national criteria prevail over international criteria for actual prescription of biologics.

This study has several limitations. First of all, our unselected cohort of CRS patients only consists of patients from tertiary hospitals. This may contribute to a selection bias of an already severe group of CRS patients. Therefore, the percentages who achieved the indication according to the recommendations might be an overestimation compared to the broader CRS population. Furthermore, enrolled patients did not provide all the necessary data using the Galenus Health application to define uncontrolled severe CRS according to the 2 recommendations.

Another limitation is that the Galenus Health application uses patient-reported outcome measures (PROMs) like visual analogue scale (VAS) scores to define anosmia. In fact, these were used as a proxy for the requirement of proven anosmia on a smell test. PROMs are easy and quick to fill out by patients for which digital technology can be useful.^23^ However, these scores are not validated for anosmia and the correlation with olfactory tests needs further investigation.^24^, ^25^, ^26^ Similar limitations apply to other proxies, such as an established diagnosis of asthma versus regular use of inhaled corticosteroids.

Ideally, a single set of criteria based on real world data should be constructed that is applicable across Europe. In practice, the local (reimbursement) situation is presently the most determining factor to whether or not patients are prescribed biologics. To start bridging this gap, European data on the cost of CRS, and on the effect of biological treatment on costs should be further investigated. Moreover, we lack data to support physicians in the choice of the biologic used; for now, there are criteria that only indicate the use of any biologic. Registries such as CHRINOSOR allow us to generate patient profiles to indicate which patients could be preferentially treated by which (biologic) therapy.

In conclusion, the EPOS/EUFOREA 2023 recommendations are more inclusive than the EUFOREA 2021 recommendation with respect to overall indication for a biologic treatment. However, actual prescription rates are similar and mostly determined by local treatment and reimbursement criteria. Obviously, this argues for a detailed cost-effectiveness analysis taking into account different aspects of (the progress of) the disease as well as patient and societal requirements.

## Abbreviations

ACT, Asthma Control Test; AMT, Appropriate Medical Treatment; app, Mobile Application; BEC, Blood Eosinophil Count; BMI, Body Mass Index; CHRINOSOR, Chronic Rhinosinusitis Outcomes Registry; CRS, Chronic Rhinosinusitis; CRSwNP, Chronic Rhinosinusitis with Nasal Polyps; EPOS, European Position Paper on Rhinosinusitis and Nasal Polyps; ESS, Endoscopic Sinus Surgery; EUFOREA, European Forum for Research and Education in Allergy and Airway Diseases; ICS, Inhaled Corticosteroids; IgE, Immunoglobulin E; INCS, Intranasal Corticosteroids; LoS, Loss of Smell (symptom score); NB, Nasal Blockage (symptom score); NERD, NSAID-Exacerbated Respiratory Disease; NPS, Nasal Polyp Score; NSAID, Non-Steroidal Anti-Inflammatory Drug; PROMs, Patient-Reported Outcome Measures; SCS, Systemic Corticosteroids; SNOT-22, Sino-Nasal Outcome Test-22; VAS, Visual Analogue Scale.

## Authorship contribution

SFS, GMa, CB designed the study. AA, IA, CC, PWH, LVG, VH, CH, AK, SS, PVT, WJF, CH, KL, SM, GMo, MM, CRvS, MdV, MW and SR recruited patients for the study. JJO, SFS, JdK and GB contributed to data analysis. JJO, SFS, JdK and SR prepared the first draft of the manuscript. All authors critically revised the manuscript.

## Ethics statement

The study was approved by the local institutional review boards, except for the Netherlands (not required according to the Dutch Medical Research Involving Human Subject Act) and Denmark (not required according to national legislation) and registered at clinicaltrials.gov (NCT04670172).

## Use of generativeAI or other AI-assisted technologies

Nothing to disclose.

## Funding

This study is funded by 10.13039/100008792Novartis Pharma AG, GSK and 10.13039/100004339Sanofi & 10.13039/100009857Regeneron.

## Conflict of interest declarations

AA: Alexandros Adrianakis: No disclosures related to this manuscript.

IA: Isam Alobid reports grants, royalties or license, consulting fees, payment for lectures, participation to advisory board from Novartis, Sanofi, Salvat, Menarini, Roche, GSK, MSD, Galenus health, Viatris, Olympus, payment for expert testimony from Teladoc.

CB: Claus Bachert reports grants or contracts from GSK, Sanofi, Novartis, Galenus Health.

GB: Giulia Bettio is an employee of Galenus Health.

CC: Carlo Cavaliere reports consulting fees from GSK, Sanofi, Novartis, Astra Zeneca, participation on advisory board from GSK, Sanofi, Novartis.

ZED: Zuzana Diamant reports consulting fees and/or payment for lectures from Sanofi-Genzyme, GSK, Galenus Health, Antabio, Arcede, Biosion, Foresee Pharmaceuticals, Hippo-Dx, Pleuran, QPS-NL, EUFOREA; leadership role in EUFOREA (asthma expert panel chair 2020–2024), associate editorships at Springer (RESNI/MedNet 2011–2025), Respiratory Medicine (2004-ongoing) and Allergy (2018–2024).

JED: Julia Eckl-Dorna reports grants (institution) from Astra Zeneca, Novartis, payment for lectures from Allergopharma, participation on advisory board from GSK, Astra Zeneca, Bencard.

WF: Wytske Fokkens reports grants (institution: AMC) from GSK, Novartis, Sanofi, consulting fees from Dianosic, Sanofi, GSK, Novartis, payment for lectures from Sanofi, GSK, Novartis, participation on advisory board from Lyra and Leadership roles in ERS (Secretary General), Rhinology (Editor in chief), Allergy (Associate Editor).

MDV: Marco de Vincentiis: No disclosures related to this manuscript.

CH: Clemens Holzmeister: No disclosures related to this manuscript.

CH: Claire Hopkins reports advisory board fees from Astra Zeneca, Dianosic, GSK, Sanofi.

VH: Valérie Hox reports payments for lectures from Novartis, participation to advisory boards of Sanofi, GSK and leadership role in EAACI.

CR: Camilo Rodriguez van Strahlen: No disclosures related to this manuscript.

PH: Peter Hellings: No disclosures related to this manuscript.

AK: Anette Kjeldsen reports grants from Astra Zeneca and participation on advisory board (until 2020) from Sanofi, board role in Danish National medical board regarding inflammatory diseases of the nose and sinuses.

JdK: Joost de Kinderen is a partner and shareholder of Galenus Health.

VL: Valerie Lund reports royalties from Elsevier, consulting fees from GSK, honoraria for lectures from Abbott, GSK, Novartis, Sanofi, support for attending meetings participation on advisory board from GSK and Sanofi.

GM: Gert Mariën is a partner and shareholder of Galenus Health.

SM: Simonetta Masieri reports consulting fees from GSK, Sanofi, Novartis, Astra Zeneca, support for attending meetings from LoFarma, Sanofi, participation on advisory board from AstraZeneca, Sanofi, Novartis.

GM: Geoffrey Mortuaire reports payments for lectures from Sanofi, SGK, Novartis, Dianosic, Medtronic, support for travel from Audika, participation in advisory boards of Sanofi, GSK, Novartis, Dianosic and board role in ALK.

SR: Sietze Reitsma reports grants (institution) from Sanofi and Novartis and consultancy fees from Sanofi, Novartis, GSK.

JO: Josje Otten: reports payment for lectures from Sanofi.

SFS: Sven Seys is an employee of Galenus Health, reports payment for lectures from Teva Pharmaceutical.

SS: Sven Schneider reports grants, payment for lectures and participation on advisory board from Sanofi, Novartis.

PVT: Peter-Valentin Tomazic reports no disclosures related to this manuscript.

LVG: Laura Van Gerven reports grants from FWO Vlaanderen and UZ Leuven, payments for lectures from ALK and participation on advisory board from Hippo Dx.

MW: Martin Wagenmann reports grants from ALK-Abello, GSK, Regeneron, Astra Zeneca, Novartis, Sanofi, Takeda, consulting fees from ALK, Genzyme, Novartis, Stallergenes, Astra Zeneca, GSK, Sanofi, payment for lectures from ALK-Abello, Astra Zeneca, GSK, Leti Pharma, Sanofi, Allergopharma, Bencard Allergie, Infectopharm, Novartis, Stallergenes, Leadership role in DGAKI (executive committee).

JM: Joaquim Mullol reports no disclosures related to this manuscript.
