# Supplementary material for: Indication for biologic treatment in a real-world cohort of chronic rhinosinusitis patients according to international recommendations: Evidence from the European CRS outcome registry (CHRINOSOR)
Source: World Allergy Organ J. 2026 Mar 23;19(4):101365. doi: 10.1016/j.waojou.2026.101365 (PMC13045673; doi:10.1016/j.waojou.2026.101365)
Supplement: Multimedia component 1 [file mmc1.docx]

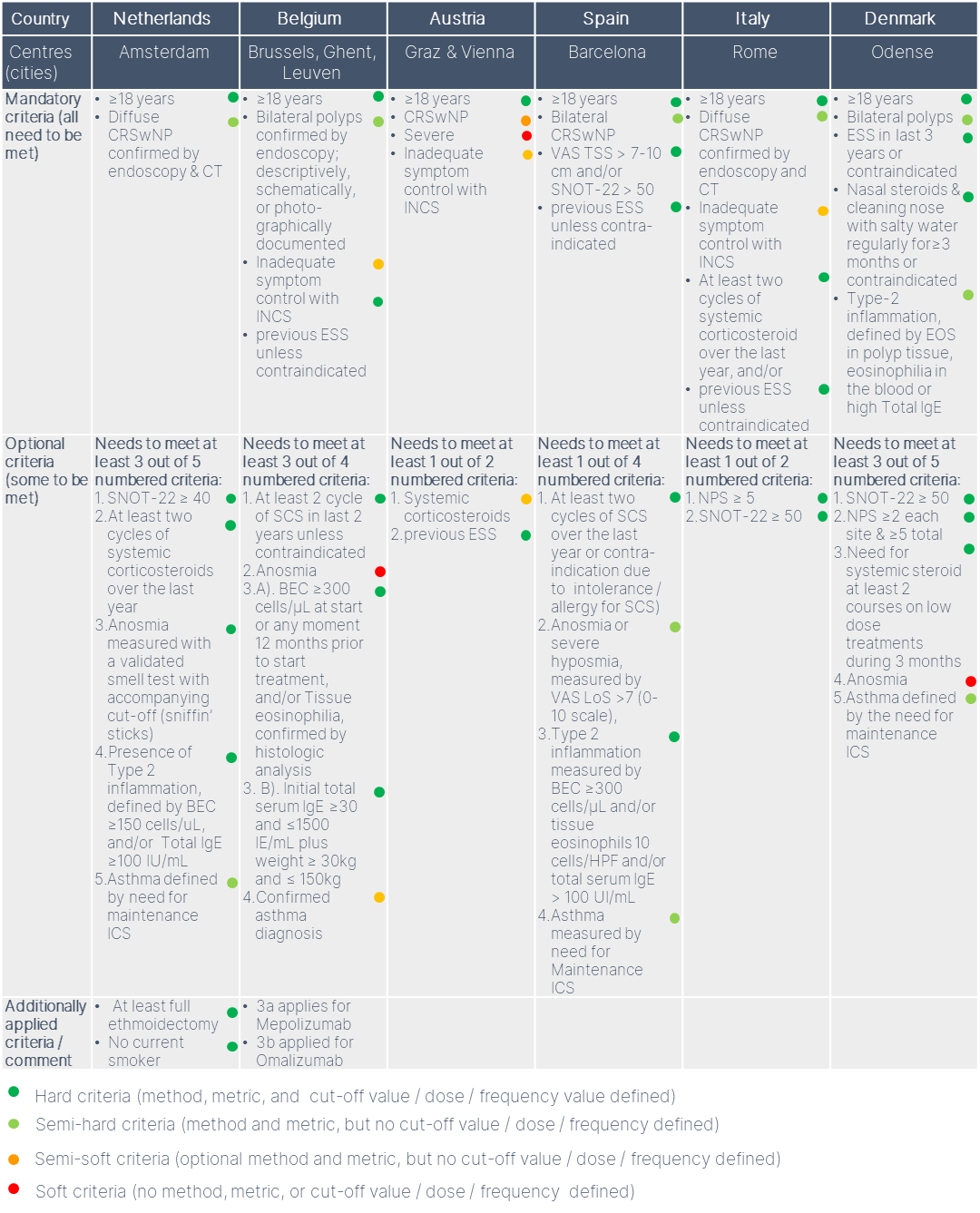


**Table S1. National reimbursement criteria biologics for bilateral CRSwNP.**

The Netherlands: [www.farmacotherapeutischkompas.nl](http://www.farmacotherapeutischkompas.nl); Belgium: [www.bcfi.be](http://www.bcfi.be); Austria: <https://medikamente.basg.gv.at/en/medicinal-products>; Spain: <https://cima.aemps.es/cima/publico/home.html>; Italy: <https://www.aifa.gov.it/en/open-data>; Denmark: <https://laegemiddelstyrelsen.dk/en>
