# Supplementary material for: Indication for biologic treatment in a real-world cohort of chronic rhinosinusitis patients according to international recommendations: Evidence from the European CRS outcome registry (CHRINOSOR)
Source: World Allergy Organ J. 2026 Mar 23;19(4):101365. doi: 10.1016/j.waojou.2026.101365 (PMC13045673; doi:10.1016/j.waojou.2026.101365)
Supplement: Multimedia component 2 [file mmc2.docx]

| Country | Centre | Ethics vote |
| --- | --- | --- |
| Austria | Blinded for review | Blinded for review |
|  | Blinded for review | Blinded for review |
| Belgium | Blinded for review | Blinded for review |
|  | Blinded for review | Blinded for review |
|  | Blinded for review | Blinded for review |
| Italy | Blinded for review | Blinded for review |
| Spain | Blinded for review | Blinded for review |

**Table S2. Ethics votes.**
